# Assessment and modeling using machine learning of resistance to scald (*Rhynchosporium commune*) in two specific barley genetic resources subsets

**DOI:** 10.1038/s41598-021-94587-6

**Published:** 2021-08-05

**Authors:** Houda Hiddar, Sajid Rehman, Berhane Lakew, Ramesh Pal Singh Verma, Muamar Al-Jaboobi, Adil Moulakat, Zakaria Kehel, Abdelkarim Filali-Maltouf, Michael Baum, Ahmed Amri

**Affiliations:** 1 Laboratory of Microbiology and Molecular Biology, Faculty of Sciences, BioBio Research Center, University Mohammed V in Rabat, Rabat, Morocco; 2 International Center for Agricultural Research in the Dry Areas (ICARDA), Biodiversity and Crop Improvement, 10010 Rabat, Morocco; 3 Berhane Lakew, Ethiopian Institute of Agricultural Research (EIAR), Addis Ababa, Ethiopia; 4 ICAR-Indian Institute of Wheat and Barley Research, Karnal, Haryana 132001 India

**Keywords:** Genetics, Plant sciences

## Abstract

Barley production worldwide is limited by several abiotic and biotic stresses and breeding of highly productive and adapted varieties is key to overcome these challenges. Leaf scald, caused by *Rhynchosporium commune* is a major disease of barley that requires the identification of novel sources of resistance. In this study two subsets of genebank accessions were used: one extracted from the Reference set developed within the Generation Challenge Program (GCP) with 191 accessions, and the other with 101 accessions selected using the filtering approach of the Focused Identification of Germplasm Strategy (FIGS). These subsets were evaluated for resistance to scald at the seedling stage under controlled conditions using two Moroccan isolates, and at the adult plant stage in Ethiopia and Morocco. The results showed that both GCP and FIGS subsets were able to identify sources of resistance to leaf scald at both plant growth stages. In addition, the test of independence and goodness of fit showed that FIGS filtering approach was able to capture higher percentages of resistant accessions compared to GCP subset at the seedling stage against two Moroccan scald isolates, and at the adult plant stage against four field populations of Morocco and Ethiopia, with the exception of Holetta nursery 2017. Furthermore, four machine learning models were tuned on training sets to predict scald reactions on the test sets based on diverse metrics (accuracy, specificity, and Kappa). All models efficiently identified resistant accessions with specificities higher than 0.88 but showed different performances between isolates at the seedling and to field populations at the adult plant stage. The findings of our study will help in fine-tuning FIGS approach using machine learning for the selection of best-bet subsets for resistance to scald disease from the large number of genebank accessions.

## Introduction

Barley (*Hordeum vulgare* L.) remains one of the most important cereal crops since its domestication in the “Fertile Crescent” 10,000 years ago^1^. It ranks fourth among cereals in terms of production and acreage and is grown mainly in Russia, Australia, Europe, United Kingdom, Canada, North America, and Asia^2^. Barley is a major crop in North Africa and West Asia, and it is grown on an average ~ 3 million ha in the dry areas of Morocco and Ethiopia^3^.

Barley is a multiple purpose crop used mainly as feed (green forage, straw, and grain), food, malt, and brewing. Due to its health benefits, it has become an important ingredient of the modern human diet in many developing as well as developed countries^4^. Particularly in Japan, more than 90% of barley is used as human food^5^. In Morocco, Ethiopia, India and China, barley grain is still widely consumed^6^. Recently, barley grain is being used in renewable energy for the production of biofuels^7^.

Barley has a great potential to adapt to a wide range of environmental conditions, ranging from desert, to high latitudes and altitudes^8^. Around the globe barley can grow in the spring and winter seasons^9^. However, barley productivity and quality are affected by several biotic factors including viruses, fungi, and bacteria^10^. Among these phytopathogens, *Rhynchosporium commune* (formerly *R. secalis*), is one of the most serious and destructive fungi causing a foliar disease known as scald or leaf blotch. This disease is found in all continents, but is more frequent and devastating under cool and semi-humid barley growing regions^11^. Yield losses due to scald can vary from 10 to 70%^11–14^. Furthermore, Home‐Grown Cereals Authority in the United Kingdom reported that despite the use of chemical fungicides, *R. commune* causes annual yield losses worth £4.8 million^4^.

Owing to its high genetic diversity and spontaneous mutations, *R. commune* populations can evolve rapidly^15^, and has the potential to quickly adapt and overcome deployed host plant resistance^16,17^, making its control more challenging. Linde et al.^18^ found 76% of the global genetic diversity of the pathogen within a single barley field. *R. commune* is an imperfect fungus, and its perfect stage has not been reported. Pathogens with mixed reproduction system pose serious threats to longevity of resistance genes. A sexual reproduction can be predicted based on equal frequency of two matting types in the population. In Turkey, Çelik Oğuz et al.^19^ reported 35% of the isolates with MAT1-1 and 65% of the isolates with MAT1-2 which hints asexual reproduction. In addition, other matting type studies indicate asexual reproduction in Iran, and Syria, while in Switzerland, Australia, Ethiopia, South Africa, Scandinavia and California, sexual reproduction has been predicted^20,21^. For developing control strategy, genetic diversity and its distribution among populations may be helpful. Furthermore*, R. commune* populations can adapt to abiotic selection pressure, comprising the variation of temperature and global warming^22^. Another challenge complicating the control of scald disease is its ability to survive, colonize and sporulate asymptomatically on the resistant varieties, which can be a source of inoculum for the next cropping season infection^23^. Many studies showed that polymerase chain reaction (PCR) can detect symptomless presence of *Rhynchosporium* in the seeds^24^, which allows the transmission of the pathogen^25^.

Virulence changes in pathogen populations affect the disease resistance status of the host plant genotypes. It is necessary to understand the evolutionary forces which impact pathogen biology in terms of emergence of individual genotypes with altered virulence. Conversely, plant genetic resources should be screened with newly emerged virulent pathotypes for effective management of the diseases. Several factors such as large population size, frequency-dependent selection, spontaneous mutation, gene flow, sexual reproduction and asexual recombination could explain the high genetic diversity of *R. commue* populations^19,26^. Different studies have reported 2–72 pathotypes based on responses of diverse isolates on selected barley genotypes^27–34^. Azamparsa and Karakaya^33^ grouped 52 scald isolates into 30 pathotypes based on the screening on 17 differential barley genotypes. None of the pathotype was virulent on all differentials and two cultivars, Jet and Abyssinia, were the most resistant with a mean disease score of 0.2–0.3 on a scale of 0–4. Therefore, a continuous search for new effective resistance genes and their deployment through breeding is needed to cope with the emerging new virulent pathotypes of leaf scald pathogen.

The use of fungicides and cultural practices are not efficient in sustainable management of leaf scald disease, and resistance to multiple fungicides has been reported by many researchers^35,36^. *R. commune* has developed insensitivity to demethylation inhibitor fungicides e.g., methyl benzimidazole carbamates^26^, and to azole-based fungicides through mutation in multiple loci^36^. Genetic resistance remains the most economical and environmentally friendly way to control scald disease^37^. Thus genetic resources, such as barley landraces and wild barley accessions, held in the gene banks are key to crop improvement efforts, as they allow continuous supply of traits sought by the breeders, including genes for resistance/tolerance to major abiotic and biotic stresses^38^. Wild barley (*Hordeum spontaneum*) offers great potential as source of resistance compared to landraces. The response of 198 landraces and 104 *H. spontaneum* accession from Turkey revealed that only 0.5% (1) of the landraces were resistant to six isolates of scald, but 26% (27) of *H. spontaneum* accessions were resistant to all isolates tested^39^. Similarly, Rehman et al.^40^ reported 58% (66) *H. spontaneum* accessions from ICARDA genebank resistant to scald under field conditions in Morocco. Silvar et al.^41^ screened 159 lines issued from a core collection of Spanish barley landraces and found 26% being resistant to scald. Yitbarek et al.^42^ found sources of resistance to scald in the Ethiopian germplasm with a higher rate of resistance in accessions originated from higher altitudes. Furthermore, van Leur et al.^43^ found 13% lines being resistant to scald from a set of 100 lines derived from landraces collected from different regions of Syria and Jordan. They observed more variation within landraces and among collection sites, with more prevalence of resistant accessions from cooler environments. Furthermore, Düşünceli et al.^44^ reported 8 out of 683 barley genotypes resistant at the seedling as well as at the adult plant stage.

ICARDA has the global mandate for the improvement of barley^45^, and it’s genebank has one of the largest collections of barley worldwide totaling around 32,800 accessions, including more than 2500 accessions of wild *Hordeum* species^46^. ICARDA distributes to collaborators the elite germplasm in the form of international nurseries and accessions of genebank based on requests and using the Standard Material Transfer Agreement (SMTA). The distribution of accessions was done in the past either using random sampling or using core collections with the latter attempting to include 90% of the diversity in 5–10% of the total holdings^47^. The Generation Challenge Program (GCP) proposed the development of a reference set representing most of the genetic diversity in 10% of the core collection using molecular markers. ICARDA with the Australian and the Russian collaborators developed an approach named “Focused Identification of Germplasm Strategy (FIGS)”, which is either based on filtering or predictive modeling, to construct best-bet subsets for targeted traits^48^. The predictive modeling approach allows to develop algorithms linking the sought plant traits to geographic, agro-climatic, and edaphic variables, which are used to construct manageable subsets for efficient evaluation and mining of genetic resources^49^. The relationship between the trait and the environment was shown in case of barley by Endersen^50^ and was tested for salinity tolerance^51^, and for net blotch resistance^52^. Furthermore, FIGS was successful in identifying sources of resistance to the Russian wheat aphid and Sunn pest in wheat which could not be identified using random sampling of larger subsets^53,54^. In addition, new alleles for resistance to powdery mildew in wheat^55^ were identified from a targeted FIGS subset.

The present study aims at the identification of sources of resistance to scald in GCP and FIGS subsets, and to assess different predictive models for efficient mining of genetic resources for resistance to scald.

## Results

### Reaction of GCP and FIGS subsets to scald disease

Scald infections were successful under both controlled and field conditions as shown by the uniformity of the infection of the susceptible accessions as well as the checks. The averages scores of susceptible (Tocada and Tissa) and resistant (ICARDA4 and Atlas 46) checks at the adult plant stage were 8 and 1, respectively. While at the seedling stage, average scores of resistant and susceptible checks for isolate SC-511 were 0 and 5, and for the isolate SC-1122 were 1 and 4, respectively.

**Figure 1:**
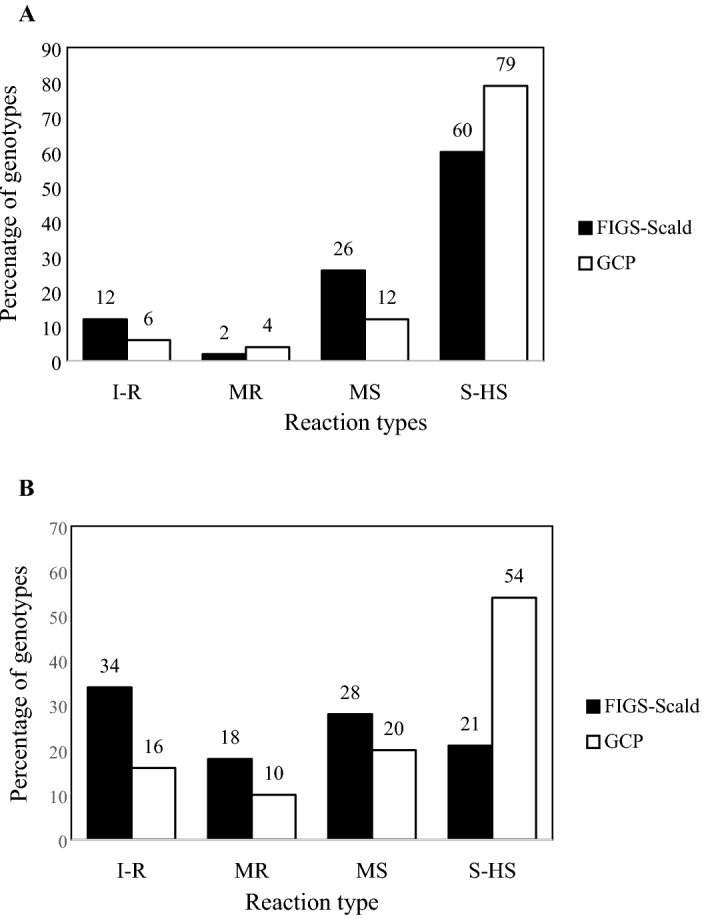
Seedling reaction to scald isolate 511 (**A**) and 1122 (**B**) of barley accessions of FIGS and GCP subsets under control conditions (in percent). Here* I–R* Immune and resistant, *MR* moderately resistant, *MS* moderately susceptible, *S-HS* susceptible and highly susceptible.

The two subsets (FIGS and GCP) included sources of resistance to scald at both the seedling and adult plant stages, but their percentages differed based on isolates at the seedling stage and based on location and pathogen populations under field conditions. At the seedling stage, GCP subset showed 6% and 16% resistant accessions, and 79% and 54% susceptible accessions to scald isolates SC-511 and SC-1122, respectively. For FIGS subset, 12% and 34% of the accessions were resistant, while 60% and 21% were susceptible to SC-511 and SC-1122 isolates, respectively (Fig. 1A,B). Different percentages for the moderately resistant and moderately susceptible classes were found among the two subsets for both scald isolates tested. FIGS subset had 8% more accessions with MR and MS reactions compared with GCP for the SC-1122 isolate. For isolate SC-511, 4% of GCP and 2% of FIGS accessions were MR while 12% and 26% showed MS reaction, respectively. Isolate SC-511 showed less percentage of accessions with immune, resistant, and moderately resistant reaction and a higher percentage of susceptible accessions compared to isolate SC-1122 (Fig. 1).

**Figure 2:**
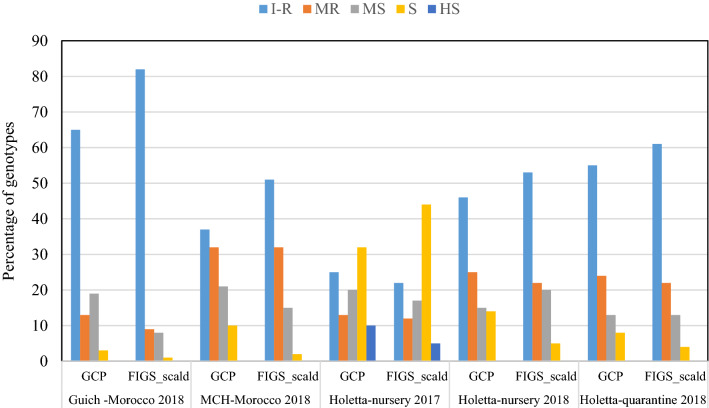
Adult plant reaction of GCP and FIGS barley subsets (in percent) to scald populations under field conditions. *I–R* Immune and resistant, *MR* moderately resistant, *MS* moderately susceptible, *S* susceptible, *HS* highly susceptible.

At the adult plant stage, the percentage of accessions for scald disease severity differed based on the location, year, and subsets. The highest percentages of resistant accessions for both subsets (65% for GCP, and 82% for FIGS) were observed at Guich-Morocco 2018, and the lowest percentages at Holetta 2017 (25% for GCP, and 22% for FIGS) (Fig. 2). For Marchouch 2018, 37% of GCP and 51% of FIGS were resistant, while for Holetta-nursery 2018, these respective percentages were 46% and 53%, and 55% and 61% for Holetta-quarantine 2018. The highest percentages of susceptible (32% for GCP, and 44% FIGS), and highly susceptible accessions (10% for GCP, and 5% for FIGS) were identified at Holetta-nursery 2017 for both subsets, while for all the other environments these classes of reaction ranged from 0 to 10%. When the disease scoring is grouped into three classes, (I + R, MR + MS, S + HS), the highest percentage of resistant accessions were found in the case of Guich Morocco-2018, and the lowest at Holetta-nursery 2017. For the moderately resistant and moderately susceptible classes, the GCP and FIGS subsets had similar percentages except at Guich-2018 where GCP (33%) had almost twice as much as FIGS (17%) (see Supplementary Fig. S1).

### Comparison of GCP and FIGS subsets

The tests of independence showed that the reaction to scald is dependent on the subsets when tested in the field at Guich and Marchouch in Morocco with χ^2^ P-values < 0.01 (Table 1). However, this dependency was not observed when the subsets were tested against the scald natural populations in Ethiopia. The same conclusions were confirmed when different classes of disease reactions were combined (see Supplementary Table S1). In case of dependency, FIGS appeared to deviate from theoretical distribution and had higher percentages of accessions with resistant and moderately resistant reactions and lower percentages of susceptible and highly susceptible accessions. At the seedling stage, the reaction types to both isolates were dependent on the subsets for all classes of reactions (P values < 0.01), except for the isolate SC-511 for the grouping (I + R + MR) and (MS + S + HS) with a p-value of 0.201 (Table 2).

**Table 1:** Test of independence of adult plant reaction of FIGS and GCP barley subsets to scald evaluated under field conditions in Ethiopia (2017–2018), and in Morocco (2018) for each disease reaction class.

Locations	Sets	Reaction type						χ^2^ (P-value)
		I	R	MR	MS	S	HS	
Guich-Morocco 2018	GCP	58	66	26	36	5	0	12.664 (P = 0.013)
	FIGS_scald	48	35	9	8	1	0	
MCH-Morocco 2018	GCP	6	64	62	40	19	0	12.099 (P = 0.017)
	FIGS_scald	8	44	32	15	2	0	
Holetta-nursery 2017	GCP	24	24	24	39	61	19	5.756 (P = 0.331)
	FIGS_scald	9	13	12	17	44	5	
Holetta-nursery 2018	GCP	38	48	47	30	26	0	6.635 (P = 0.156)
	FIGS_scald	26	27	22	20	5	0	
Holetta-quarantine 2018	GCP	42	61	46	25	15	0	5.231 (P = 0.264)
	FIGS_scald	33	27	22	13	4	0	

*I* Immune, *R* resistant, *MR* moderately resistant, *MS* moderately susceptible, *S* susceptible, *HS* highly susceptible.

**Table 2:** χ^2^ tests of independence and of goodness of fit of different groups of disease reaction classes to two scald isolates (511, 1122) under controlled conditions using GCP and FIGS subsets of barley.

Isolates	Set	Group of 6 classes	Group of 2 classes	Group of 3 classes
		χ^2^ (P-value)	χ^2^ (P-value)	χ^2^ (P-value)
**Test of independence**				
SC-511	GCP	21.352 (< 0.01)	1634 (P = 0.201)	12.107 (< 0.01)
	FIGS_scald			
SC-1122	GCP	35.140 (< 0.01)	18,265 (< 0.01)	30.290 (< 0.01)
	FIGS_scald			
**Test of goodness of fit**				
SC-511	GCP	39.944 (< 0.01)	2.851 (P = 0.091)	22.143 (< 0.01)
	FIGS_scald			
SC-1122	GCP	60.501 (< 0.01)	18.265 (< 0.001)	46.698 (< 0.01)
	FIGS_scald			

Group of 6-classes includes reaction types I, R, MR, MS, S, HS, group of 2-classes include I + R + MR and MS + S + HS, group of three classes include I + R, MR + MS, and S + HS.

*FIGS* Focused Identification of Germplasm Strategy subset, *GCP* Generation Challenge Program reference set.

For the test of goodness of fit, highly significant differences between FIGS and GCP subsets were declared based on a comparison of different classes of disease reactions at Guich and Marchouch (2018) in Morocco with P values < 0.01. These differences were significant (P-value = 0.033) in case of the trial in Holetta-nursery 2018, but not in Holetta-nursery 2017 (see Supplementary Table S2). When the differences were significant, FIGS subset showed higher percentages of resistant classes and lower percentages of susceptible classes than GCP subset at the adult plant stage. When the tests were done at the seedling stage using both isolates, the differences were highly significant between FIGS and GCP subsets for all groups of disease reaction classes except for the isolate SC-511 for the grouping (I + R + MR) and (MS + S + HS) with a p-value of 0.091 (Table 2).

### Prediction of reaction to scald using machine learning approach

In our present study the performance of predictive models varied depending on the environment at the adult plant stage, and on the isolates at the seedling stage. At the seedling stage, the two modeled classes were not balanced for both high and averages scores (Table 3). The isolate SC-1122 showed a better balance than isolate SC-511 between resistant and susceptible classes. High score exhibited a higher but non-significant accuracy than the average score for both isolates. However, models for average accuracy were significantly higher than Null accuracy with 0.89 and 0.64 for isolate SC-511 and SC-1122, respectively. Models for both isolates, and both scores were not able to identify efficiently resistant accessions due to low sensitivity with maximum value of 0.25.

**Table 3:** Modeling performance from the best machine learning algorithm for seedling data for high (HS) and average (AS) disease scores for scald disease in barley set.

Best model	SC-511 HS	SC-511 AS	SC-1122 HS	SC-1122 AS
	BCART	BCART	KNN	SVM
Sensitivity	0.20	0.11	0.25	0.25
Specificity	0.96	1.00	0.84	0.88
Precision	0.25	1.00	0.36	0.54
Accuracy	0.91	0.89	0.68	0.64
Kappa	0.17	0.18	0.10	0.14
Accuracy lower	0.82	0.80	0.57	0.53
Accuracy upper	0.96	0.95	0.79	0.75
Accuracy null	0.93	0.88	0.74	0.63
Mcnemar (P-value)	1.00	0.01	0.31	0.01

*BCART* Bagged Carts, *KNN* K-nearest neighbors, *SVM* support vector machine.

At the adult plant stage for the combined field data, only two models showed significant and accurate results, BCART and RF with an accuracy of 0.73 and 0.72, respectively (see Supplementary Table S3). Specificity was very low-to-medium for all the four assessed models, and the Null accuracy was higher than 0.5 and reached to 0.67. All models efficiently identified resistant accessions with specificities higher than 0.88. Modeling of field data per location/environment (Table 4) showed different patterns. Accuracy from modeling Marchouch was significantly higher than Null model with an accuracy of 0.81 and Kappa of 0.53.

**Table 4:** Performance metrics for the best machine learning models for scald reaction of barley at adult plant stage in different environments.

Best model	GUICH	MCH	Holetta17	Holetta18
	BCART	RF	SVM	KNN
Sensitivity	0.94	0.95	0.41	0.73
Specificity	0.23	0.53	0.77	0.25
Precision	0.86	0.81	0.52	0.63
Accuracy	0.82	0.81	0.63	0.55
Kappa	0.21	0.53	0.19	− 0.02
Accuracy lower	0.72	0.75	0.51	0.43
Accuracy upper	0.90	0.87	0.74	0.67
Accuracy null	0.83	0.69	0.62	0.63
Accuracy (P-value)	0.69	0.00	0.46	0.94

*BCART* Bagged Carts, *RF* Random forest, *SVM* support vector machine, *KNN* K-nearest neighbors, *MCH* Marchouch station.

## Discussion

The good development of scald under natural infection in Holetta-Ethiopia and under artificial inoculation with the use of overhead sprinklers irrigation system in Morocco allowed reliable screening of FIGS and GCP subsets. Over the two cropping seasons, scald appeared as a permanent threat to barley in Ethiopia while in Morocco its natural development was more sporadic in time and space. The surveys conducted in North African countries during 1990–1994 through UNDP-ICARDA project on cereals and food legume diseases showed that scald is a major disease of barley in Tunisia, but in Morocco, infections were observed only once out of five years (unpublished data). Epidemics were observed in 1998 in Eritrea, Ethiopia, Turkey, Tunisia, and Morocco^56^. During 2018 and 2019, the visits of farmers’ fields in many regions of Morocco showed a low incidence of scald with fields showing medium severity including in mountainous and semi-arid regions (unpublished data). All released varieties in Morocco are moderately to highly susceptible to scald^57^, and host plant resistance was chosen as the most-appropriate approach for integrated management of the disease.

Our results showed that resistance was found in both FIGS and GCP subsets at the seedling and adult plant stages. The reaction to the disease differed between isolates, seasons, field locations, and between subsets at both stages. The differences in reactions could be attributed to virulence differences among the isolates at the seedling stage with isolate SC-511 being more aggressive than isolate SC-1122, and to differences in virulence spectra among scald field populations at the adult plant stage (Fig. 1). A similar finding was reported by Albustan et al.^58^, where they found 31% resistant genotypes at the adult plant stage and 19% at the seedling stage.

Although sources of resistance to scald were identified in both subsets at both stages, in general FIGS subset presented higher percentages of resistant accessions (54%) at both stages compared to GCP subset (45%). Likewise, 26% accessions in a Spanish barley core collection^41^, and 13% landraces from a set of lines derived from Syrian and Jordanian landraces^43^ were resistant to scald. Our study is the first attempt to compare FIGS to another subset, and the results are supporting the efficiency of this approach in mining genebank collections. Several other studies have shown the limitation of the core collection approach for identifying and searching for rare and adaptive alleles^59–61^. In addition, the core collection and the GCP reference set derived from it are targeting higher overall diversity of the accessions, while FIGS is targeting the diversity for resistance to scald. However, more research is needed to assess the efficiency of both GCP and FIGS of finding different effective genes for adaptive traits such as resistance to scald. Furthermore, genotyping and association mapping studies could decipher if resistance is conditioned by new resistance genes or by new allelic variants of the existing resistance genes.

The high likelihood of containing the targeted alleles in FIGS-subset is most probably due to the co-evolutionary process that occurs over time between the host and the pathogen, and which might allow the maintenance of a high number of effective avirulence genes in the pathogen populations. Several studies demonstrated that the environment has a great influence on the development of the disease as well as the genetic structure of both the host and pathogen through processes of natural selection, gene flow, and spatial/geographic differentiation^62,63^. The filtering approach of FIGS was based on environmental conditions best suited for the natural development of the disease and this approach might not be applicable to the favorable areas where improved varieties are used at a large scale, and which has affected the diversity of the pathogen populations by continuous selection of more virulent races. In addition, our results support to some extent the theoretical basis of FIGS approach and presents further evidence that the geographical distribution of the disease resistance in nature is not random, and it is influenced by environmental conditions and the host diversity. However, our FIGS modeling approach using machine learning did not show a strong relationship between resistance to scald and environmental condition for all isolates and field populations. This could be due to the limited size of the sets, and the unbalanced distribution of reaction to scald. In addition, this could also be a result of the variability within a collection site^43^, or a limited correlation between disease resistance and environmental conditions as Silvar et al.^41^ reported that resistance was found across the country. However, more resistance was found in winter types in Spain and at higher altitudes of Ethiopia^42^. This geographic structure of disease resistance distribution was also evidenced for stem rust in wheat^64^, and powdery mildew in barley^65^.

Several previous studies using FIGS approach in different crops supported the evidence of identification of desirable expression traits using eco-geographical data^53–55^. FIGS approach was successfully used to identify source of resistance to stem rust and powdery mildew in wheat^55,66,67^, and net-blotch in barley^52^. The superiority of FIGS filtering approach was not confirmed at Holetta-nursery 2017 in Ethiopia, showing the need for refining the sub-setting process through consideration of the virulence spectra of the pathogen populations. The filtering approach can also be refined or improved further by the search of the best predictive models that can explain the relationship between the resistance and agro-climatic conditions using machine learning.

Using single isolates at the seedling stage, and the use of natural and artificial infection under the field conditions, the results showed that the reactions of both FIGS and GCP subsets to *R. commune* population are highly variable. The instability of responses could be attributed to the variable nature of the *R. commune* pathogen in terms of pathogenicity, genetic makeup of barley genotypes grown in a region, and response to environmental factors. The major resistance gene will exert selection pressure on virulence allele frequency of the pathogen isolates which will eventually break down the deployed resistance within a short span^68^. Selection is one of the evolutionary factors which can be controlled to some extent by human interventions to slow down the evolution of genetic variants with altered pathogencity. Mert and Karakaya^69^ found 7 barley cultivars resistant to all five *R. commune* isolates from Turkey. In another study, Azamparsa et al.^70^ treated a barley cultivar (Tokak 157/37) with gamma irradiation and tested 25 advanced mutant lines with three virulent isolates of scald from Turkey. Though Tokak 157/37 was highly susceptible to three *R. commune* isolates, two mutant lines were resistant to at least two isolates tested. This approach is suitable to introduce resistance in popular barley cultivars which are difficult to replace. Similar findings of high variability of the pathogenicity of the *R. commune* were also reported across the world^26,33,71^. All studies suggest complex interaction of scald isolates with barley genotypes and determination of pathotype diversity is essential to develop effective screening protocols.

The observed variability in virulence in *Rhynchosporium* is probably due to the high genetic variation of the pathogen population as reported by several studies^72,73^. The use of molecular markers showed limited genetic diversity in scald isolates from West Asia and North Africa^74,75^, however, high genetic variation was reported from Australia, California, Finland, and Norway *R. commune* populations^76,77^. But a clear correlation between molecular markers and pathogenicity of scald populations is lacking which indicate the involvement of other molecular mechanisms which drive the evolution of virulence in *R. commune*. In general, virulence genes are located on chromosomal regions rich in transposable elements which are more prone to recombination/mutation providing an evolutionary advantage to the pathogen to come up with novel variations in its effector repertoire to evade host defenses^78^. The study of the evolution of necrosis-inducing protein (NIP1) effector from 146 strains of four *Rhynchosporium* spp. revealed its presence in all species, but presence/absence polymorphism in addition to copy number variation in *R. commune* revealed a strong selection pressure on this effector protein^79^. The deployment of major resistance genes in rotation along with prudent use of fungicides can reduce the selection of virulent isolates and enhance the longevity of deployed resistant cultivars^80^.

The Focused Identification of Germplasm Strategy (FIGS) based on filtering agro-climatic conditions favoring the development of the disease has shown its efficiency in identifying a higher frequency of resistant accessions compared to the Reference Set of the Generation Challenge Program (GCP). However, more refinement of this approach is needed to take into consideration the specter of virulence of the pathogen and the development of best-bet models linking resistance to environmental conditions using machine learning.

## Methods

### Germplasm used

A total of 292 genotypes from the ICARDA genebank were evaluated for scald resistance including 101 accessions selected using FIGS approach (FIGS subset), and 191 accessions from the Generation Challenge Program reference set (GCP).

The filtering approach was used to develop FIGS subset based on the following parameters:

- Count number of days where the average daily temperature is between 8 and 12 °C, 10 days before the onset of the growing period and up to 15% into the vegetative phase.
- Remove sites with zero count from step 1.
- Sum daily rain for 10 days before the onset of the growing period up to 10% into the vegetative phase.
- Normalize both variables (steps 1 and 2) to range 0–1 for each site.
- Add variables to create index 1.
- Rank based on index 1 and remove the bottom 25 percent of sites.

For the remaining sites, the following was done:

- From 10% into the vegetative phase until onset of grain filling divide into 3 separate sub-phases of equal length.
- For each sub-phase count the number of days where the average daily temperature is between 15 and 20 °C.
- For each sub-phase determine the amount of precipitation.
- Remove sites if any of the variables = 0 (3 count variables and 3 precipitation variables).
- Normalize each variable for a range between 0 and 1.
- Add each variable and then add index 1 to create index 2.
- Rank sites using index 2 from largest to smallest.

Depending on the required subset size and the number of remaining candidate sites, proceed with the selection of accessions.

- If there are fewer candidate sites than the required set size, choose one accession in a cyclic manner working down the rank and then repeat starting at the top of the rank with sites that still have accessions after each pass. Alternatively, starting from the top ranked site, multiple accessions per site could be chosen until desired set size is achieved.
- If there are more sites than the desired set size, then one accession could be chosen randomly from each site starting at the top ranked site until the desired set size is reached. Alternatively, this approach could be taken after one candidate accession is donated by each country represented in the candidate site list.

### Fungal isolates and inoculation

Scald infected barley leaves were collected during the disease surveys conducted from 2015 to 2018 in naturally infected barley fields from different agro-ecological zones of Morocco including Marchouch and Sidi Allal Tazi research stations. Infected barley leaves were dried in paper envelops at room temperature for 4–5 days before their storage at 4 °C till further use. Air dried leaves were cut into 2 mm segments and soaked in sterile distilled water for 15 min followed by surface sterilization using 50% ethanol for 15 s and 10% sodium hypochlorite solution for 30 s, rinsed with sterile distilled water three times, and dried up within two layers of sterile Whatman filter paper. Then the leaf segments were incubated on Lima Bean agar (LMA) supplemented with Kanamycin and Streptomycin (50 mg per liter) at 14 °C in darkness for two weeks until the sporulating white or pink colonies were visible. A total of 50 single conidial isolates were prepared and stored at—80 °C freezer for long term storage. Based on race analysis, 12 pathotypes were revealed and two scald isolates with a wide virulence spectrum (Unpublished data), Marchouch (MCH-SC) and Sidi Allal Tazi (SAT-SC), were used for seedling screening in the greenhouse. While for adult plant screening, inoculum composed of a mixture of 15 pathotypes from different agro-ecological zones of Morocco was used.

### Seedling screening (SRT)

About 4–5 seeds of each accession were sown in peat moss supplemented with 14-14-14 NPK in a single cone of 3.8 cm diameter and 14 cm depth (Stuewe & Sons, Inc., Oregon, USA) in two replications. The seedlings were grown in a growth chamber at a photoperiod of 16 h light/8 h dark at 20 ± 1 °C. At the two leaves stage, inoculation was performed separately with two virulent isolates of barley leaf scald (SC-511, and SC-1122). The inoculum was prepared from 12 to 14 days old LBA plates by rubbing the agar surface gently followed by filtration with two layers of cheese cloth. Spore suspension of 5 × 10^5^ conidia/ml supplemented with surfactant Tween 20 (0.01%) was sprayed with a hand held sprayer till run off^81^. The inoculated seedlings were kept at 100% relative humidity in dark in the growth chamber for 72 h at 15 °C to enhance spore germination. Afterward, the seedlings were transferred to the greenhouse with a light/dark regime of 16/8 h at 16 ± 1 °C, respectively.

The disease evaluation at the seedling stage was carried after 16 days post-inoculation. Assessment of plant infection type and severity was done based on scoring scale (0–5) of Salamati and Tronsmo^82^ considering lesion morphology, size, chlorosis and necrosis. Where 0 = Immune (I), 1 = resistant (R), 2 = moderately resistant (MR), 3 = moderately susceptible (MS), 4 = susceptible (S), and 5 = highly susceptible (HS). For further analysis, the genotypes were categorized into either resistant (IR of 0, 1, 2) or susceptible (IR > 3, 4, 5) group.

### Adult plant screening (APS)

The adult plant screening was conducted in Ethiopia and Morocco. In Morocco, the trials were conducted at the INRA experimental stations of Marchouch (MCH; 33° 56 N, 6° 63 W) and at Guich (33° 58 N, 6° 51 W) during the cropping season of 2018. Each entry was seeded in 1 m length row with a row spacing of 0.5 m in an augmented design. Two susceptible (Tocada and Tissa) and resistant (ICARDA4 and Atlas 46) checks were planted after every 10 accessions and each block was surrounded by a perpendicular border row, composed of a mixture of scald susceptible genotypes (Tiddas, Aglou, Tissa, Adrar, Shepherd, Fleet, Baudin and Alester), to maintain high disease pressure. Artificial field inoculations were performed four times starting from Zadoks scale^83^ GS30 at an interval of 10–12 days apart in the evenings with inoculum composed of spore suspension composed of a mixture of 50 *R. commune* isolates collected from different agro-ecological zones of Morocco using a knapsack sprayer. In addition, scald infected barley residues were spread uniformly in the field. Following inoculations, the covering of spreader rows with a plastic sheet overnight and the frequent use of a mist system with overhead sprinklers ensured good disease establishment and uniform spread of the disease. In Ethiopia, the experiment was conducted in Holetta experimental station (9° 00′ N and 38° 30′ E) during 2017 and 2018 cropping seasons using the same design as described above. There, the environmental conditions were favorable to allow a high natural infection level of scald for efficient screening of the germplasm. The disease severity was assessed at GS 73–75 using 0–9 scale^84^. Genotypes were classified in the following categories: Resistant (0–2), moderately resistant (3–4), moderately susceptible (5–6), susceptible (7–8), and highly susceptible (9).

### Data analysis

The statistical analysis was performed using R software^85^. The disease assessment data from the seedling and adult plant stage were tested for statistical association between the sub-setting approaches using χ^2^ tests of independence with significance level (*α* = 0.05). The equation used to calculate chi-square is as follows:

1 $${\chi }^{2}= \sum_{i=1}^{r}\sum_{j=1}^{c}\frac{{({O}_{ij}-{E}_{ij})}^{2}}{{E}_{ij}}.$$

The equation for calculating expected values in a test of independence is as follows:

2 $${E}_{ij}= \frac{\sum_{k=1}^{c}{O}_{ij}\sum_{k=1}^{r}{O}_{kj}}{N},$$

where is the ${E}_{ij}$ = expected value, $\sum_{k=1}^{c}{O}_{ij}$ is the Sum of the ith column, $\sum_{k=1}^{r}{O}_{kj}$ is the Sum of the kth row, N is the total number.

The expected values for the test of goodness were calculated using following equation:

3 $${E}_{i}=n{p}_{i},$$

where E_i_ is the expected value, n is the total sample size, and p_i_ is the hypothesized proportion of observations in level *i*.

To find out the differences between FIGS and GCP subsets in terms of reaction to scald, the test of goodness of fit using χ^2^ test at a significance level (*α* = 0.05) was used where GCP was simulated to a random sample. Both tests were done using different groupings of reactions, all six classes (I, R, MR, MS, S and HS), three classes (I + R, MR + MS, S + HS) and two classes (I + R + MR and MS + S + HS).

### Modeling of the reaction to scald disease

The machine learning approach using the obtained SRT and APS disease screening data was used to find out the best models that link adaptive traits, environments (and associated selection pressures) with genebank accessions. We used environmental data from WorldClim1 databases as predictors. WordClim is an open access database providing global climatic layers describing past climatic profiles of collection sites and intended for spatial modeling or mapping.

It includes average, monthly minimum and maximum temperatures, precipitation, and bioclimatic variables^86^.

The following machine learning algorithms were used: K-nearest neighbors (KNN)^87^, Support Vector Machines (SVM)^88^, Random Forest (RF)^89^, and Bagged Carts (BCART)^90^. Each machine learning model was tuned to select best tuning parameters using a training set (70% of the total set) and then the best model was selected between different machine learning models based on several metrics including accuracy, specificity, and Kappa. The modeling metrics were computed on the test set (30% of the total set). In this study, R language and caret library were used for machine learning^91^. Models were tuned for parameter’s optimization and trained on 70% of the data and tested with 10 cross validation folds and 100 replications. Modeling was done for the two isolates for the seedling stage using average and high scores separately. For the adult plant reaction, modeling was done for the combined multi-locations data sets and for each location separately.

### Statements of compliance

The corresponding author attests that: The plants were regenerated and handled using the standard operating procedures set by ICARDA genebank following the FAO genebank standards. All accessions used in this study were acquired from ICARDA genebank in accordance with the International Treaty on Plant Genetic Resources for Food and Agriculture.

### Statements of consent

The corresponding author attests that: The seeds and plants were planted and evaluated with the consent and mutual understanding between ICARDA genebank and the Ethiopian Institute of Agricultural Research (EIAR). Standard operating procedures set by ICARDA genebank and EIAR were followed. We did not collect any barley leaves for this study. All the barley accessions were obtained from ICARDA genebanks using Standard Material Transfer Agreement.

## Supplementary Information

Supplementary Information.

## Data Availability

The data that support the findings of this study are stored in the ICARDA genebank database and can be made available upon request from the corresponding author.
