# Supplementary material for: Assessment and modeling using machine learning of resistance to scald (Rhynchosporium commune) in two specific barley genetic resources subsets
Source: Sci Rep. 2021 Aug 5;11:15967. doi: 10.1038/s41598-021-94587-6 (PMC8342473; doi:10.1038/s41598-021-94587-6)
Supplement: Supplementary file 1 — Supplementary Information. [file 41598_2021_94587_MOESM1_ESM.docx]

**Supplementary Table S1:** Test of independence of adult plant reaction of FIGS and GCP barley subsets to scald evaluated under field conditions in Ethiopia (2017-2018), and in Morocco (2018) based on different grouping of the disease reaction classes.

| Locations | Sets | Group of 2 classes | Group of 3 classes | |
| --- | --- | --- | --- | --- |
|  |  | **χ^2^ (P-value)** | **χ^2^ (P-value)** | |
| Guich -Morocco 2018 | **GCP** | 7.338 (< .01) | 9.592 (< .01) | |
|  | **FIGS_Scald** |  |  |  |
| MCH-Morocco 2018 | **GCP** | 6.782 (< .01) | 9.923 (< .01) | |
|  | **FIGS_Scald** |  |  |  |
| Holetta-nursery 2017 | **GCP** | 0.387 (P = .534) | 1.347 (P = .510) | |
|  | **FIGS_Scald** |  |  |  |
| Holetta-nursery 2018 | **GCP** | 0.695 (P = .405) | 5.464 (P = .065) | |
|  | **FIGS_Scald** |  |  |  |
| Holetta-quarantine 2018 | **GCP** | 0.591 (P = .442) | 2.010 (P = .366) | |
|  | **FIGS_Scald** |  |  |  |
| Group of 2-classes includes reaction types I+R+MR and MS+S+HS, group of three classes include I+R, MR+MS, and S+HS. | | | |  |

**Supplementary Table S2:** **χ^2^** tests of goodness of fit of different groups of disease reaction classes to scald under field conditions in Morocco and Ethiopia using FIGS and GCP subsets of barley.

| Locations | Group of 6 classes | Group of 2 classes | Group of 3 classes |
| --- | --- | --- | --- |
|  | **χ^2^ (P-value)** | **χ^2^ (P-value)** | **χ^2^ (P-value)** |
| Guich -Morocco 2018 | 18.646 (< .01) | 9.444 (< .01) | 53.610 (< .01) |
| MCH-Morocco 2018 | 18.853 (< .01) | 9.350 (< .01) | 13.255 (< .01) |
| Holetta-nursery 2017 | 8.642 (P = .124) | 0.582 (P = .446) | 2.080 (P = .353) |
| Holetta-nursery 2018 | 8.807 (P = .066) | 1.028 (P = .311) | 6.848 (P = .033) |
| Holetta-quarantine 2018 | 8.344 (P = .080) | 0.856 (P = .355) | 2.700 (P = .259) |
| Group of 6-classes includes reaction types I, R, MR, MS, S, HS, group of 2-classes include I+R+MR and MS+S+HS, group of three classes include I+R, MR+MS, and S+HS. | | | |

**Supplementary Table S3: C**omparison of modeling performance between four machine learning algorithms for the combined field data of reaction to scald in all barley accessions tested.

| Best model | BCART | KNN | RF | SVM |
| --- | --- | --- | --- | --- |
| Sensitivity | 0.88 | 0.91 | 0.89 | 1.00 |
| Specificity | 0.43 | 0.26 | 0.39 | 0.00 |
| Precision | 0.76 | 0.71 | 0.74 | 0.67 |
| Accuracy | 0.73 | 0.69 | 0.72 | 0.67 |
| Kappa | 0.34 | 0.19 | 0.30 | 0.00 |
| Accuracy Lower | 0.69 | 0.65 | 0.68 | 0.62 |
| Accuracy Upper | 0.77 | 0.73 | 0.76 | 0.71 |
| Accuracy Null | 0.67 | 0.67 | 0.67 | 0.67 |
| Accuracy (P-Value) | 0.00 | 0.15 | 0.01 | 0.52 |
| BCART, Bagged Carts; KNN, K-nearest neighbors; RF, Random forest; SVM, Support vector machine | | | | |

**Supplementary Figure S1.** Adult plant reaction of GCP and FIGS barley subsets (in percent) to scald populations under field conditions when grouped into 3 classes (I-R: Resistant class; MR-MS: Moderate class; S-HS: Susceptible class).
